# Moisture transverse moving mechanism during presteamed oak lumber drying

**DOI:** 10.1038/s41598-019-54430-5

**Published:** 2019-12-03

**Authors:** Chengyuan Li, Chun-Won Kang, Xue-Feng Zhao

**Affiliations:** 1 Department of Wood Science and Engineering, Beihua University, Room 113, Yifu building, No. 3999, Binjiang East Road, Jilin City, Jilin Province 132013 P.R. China; 2 Department of Housing Environmental Design, and Research Institute of Human Ecology, College of Human Ecology, Chonbuk National University, Room 504, Building No. 7-1, 567 Baekje – daero, Deokjin-gu, Jeonju-si, Jeollabuk-do Republic of Korea; 3 Department of Wood Science and Engineering, Beihua University, Room 113, Yifu building, No. 3999, Binjiang East Road, Jilin City, Jilin Province 132013 P.R. China

**Keywords:** Biomaterials, Tissues, Structural materials

## Abstract

Effect of steaming at 100 °C, 80 °C and 60 °C dry-bulb temperature and 0 °C wet-bulb depression for 4 hours prior to drying on the drying rate and drying deformation of 25.4 mm thick oak lumbers during kiln drying was explored in this study. The results showed that presteaming delayed the drying time by at least 19 percent, and increased the crooks and bows of the lumbers. The mechanism that presteaming delayed the drying time is attributed to the smaller and fewer moisture transverse pathways inside the surface layers of presteamed lumbers. These pathways decrease the moving rates of the bound water and the water vapor from the inner part to the surface layers of presteamed lumbers during the middle and last stage of drying because of reduced distance between the microfibrils and increased crystallinity.

## Introduction

Steaming of wood has been practiced in Europe as a preparation for drying since the middle of the 18^th^ century. Various reasons have been put forward by kiln operators to justify the steaming of green lumber. These reasons include sterilization, color improvement, accelerated drying, reduction in drying degrade, improvement in machining properties, and wood stability^1^. One of most controversial research results has been effect of presteaming on the drying time and drying defect of wood since a long time in the field of wood drying in the world. At least half of the energy used for solid wood processing was consumed during presteaming and wood drying stages. Steaming solid wood prior to kiln drying not only consumed more than half of the whole steam in wood drying, but also was related to drying time and drying defect. To save energy and natural resources, it is necessary to dry wood in the shortest time possible using the least amount of steam and drying defects.

Most researchers^1–6^ have affirmed that presteaming shortened the drying time and decreased the drying defect of wood. Few researchers proposed some different opinions. Avramidis and Oliveira^7^ concluded that no clear effect of presteaming on the drying rate of hem-fir lumber when compared to the control. Harris *et al*.^8^ reported that the drying rate of presteamed oak lumber was higher during the initial stages of drying, however, in the latter two-thirds of the drying cycle, there was very little difference in drying rate between presteamed and unsteamed oak lumber. Chafe and Ananias^9^ found that the effect of presteaming on green boards of Eucalyptus regnans was generally to reduce drying rate, and to increase checking in radial direction. However, all researchers did not reveal the mechanism that presteaming delays the drying time of wood, which **is** related to the moisture transverse moving mechanism during wood drying. For over two decades, we have operated a large scale production practice that guides kiln drying for hundreds of runs of hardwood lumbers. We confirmed that presteaming delayed the kiln drying time by about 15–20 percent, depending on the species and thickness of the lumber, and also increased hardwood lumber’s drying defects by about 5 percent compared to the control. However, the mechanism for this is unknown. This study was conducted to investigate effect of presteaming oak lumber at 100 °C 80 °C and 60 °C on the drying rate and drying deformation of oak lumbers during kiln drying, and to reveal the mechanism that presteaming delays the drying time.

## Results

25.4 mm thick oak lumbers were kiln dried after steamed at 100 °C 80 °C and 60 °C dry-bulb temperature and 0 °C wet-bulb depression for 4 hours in order to determine effect of presteaming on the drying rate and drying deformation of the lumbers. And the permeability during drying and the crystallinity after drying inside the surface layers of lumbers were measured in order to ascertain moisture transverse moving mechanism during drying in this study. The results of this study were as following. Presteaming delayed the drying time by at least 19 percent because the fewer and smaller moisture transverse pathways inside the surface layers of presteamed lumbers decrease the moving rates of the bound water within the cell walls and the water vapor in the lumens from the inner part to the surface layers of presteamed lumbers during the middle and last stage of drying, owing to reduced distance between the microfibrils and increased crystallinity. Presteaming significantly increased the crooks and bows of the lumbers because of the larger compressive stresses after stresses reversed.

## Discussion

### Permeability

**Figure 1:**
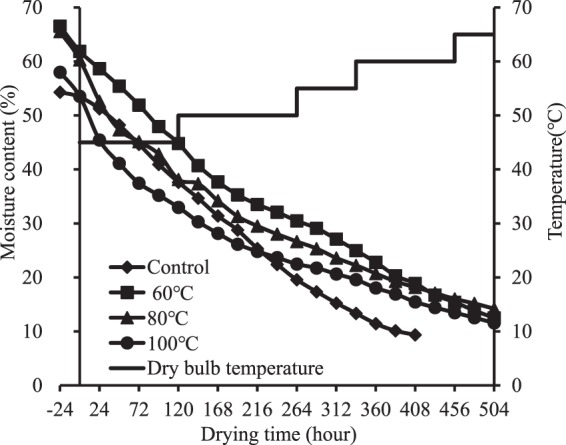
The tangential permeability of the surface layers of specimens at different stag of drying.

The result of F-test for permeability indicated that there was significant difference in the permeability among the specimens presteamed at 60 °C, the specimens presteamed at 80 °C, the specimens presteamed at 100 °C and control specimens at the initial, middle and last stage of drying (p < 0.0001). As seen in Fig. 1, the permeability in the surface layers of presteamed specimens was lower than that in control specimens, and tended decrease as presteaming temperature increased. This tendency implies that the moisture transverse pathways in the surface layers of presteamed specimens become smaller and/or fewer compared to control specimens, and which increases as presteaming temperature increased.

### Relative crystallinity

**Figure 2:**
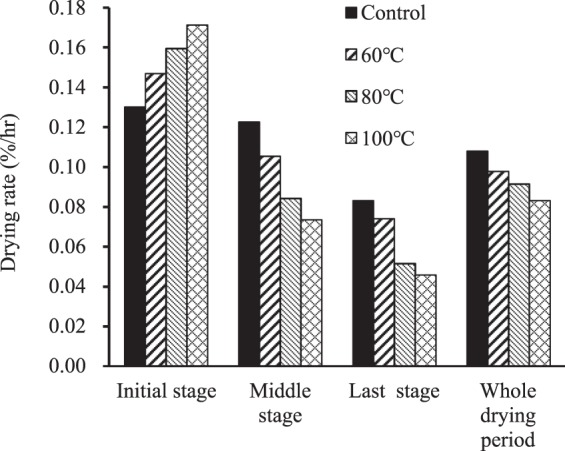
Relative crystallinity of the surface layers of specimens for different treatment.

The statistical analysis for relative crystallinity showed that there was significant difference in relative crystallinity among the specimens presteamed at 60 °C, the specimens presteamed at 80 °C, the specimens presteamed at 100 °C and control specimens through one-way analysis of variance (p < 0.0001). Comparison by LSD method indicated that difference in relative crystallinity between any two groups of specimens among the specimens presteamed at 60 °C, the specimens presteamed at 80 °C, the specimens presteamed at 100 °C and control specimens was significant. And the result of comparison by Tukey method was the same as that of LSD method. As seen in Fig. 2, the relative crystallinity in the surface layers of presteamed specimens was more than that in control specimens, and tended increase as presteaming temperature increased. This tendency implies that there is more crystalline region, which means that there is less amorphous region, in the surface layers of presteamed specimens compared to control specimens.

### Drying rate

**Figure 3:**
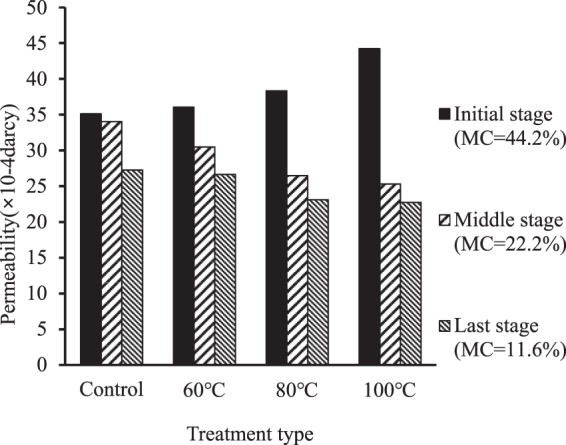
Average drying rate during whole period and the drying rates of specimens at different drying stage.

The F-test of drying rate indicated that there was significant difference in average drying rate during whole drying period (p < 0.0003) and during initial (p < 0.0001), middle (p < 0.0001) and last drying stage (p < 0.0001), respectively, among the specimens presteamed at 60 °C the specimens presteamed at 80 °C the specimens presteamed at 100 °C and control specimens. As seen in Fig. 3, the average drying rate of presteamed specimens was slower than that of control specimens during whole drying period, and tended decrease as presteaming temperature increased. This tendency implies that the microstructure and components in the surface layers of presteamed specimens were changed during presteaming and subsequent drying process.

During the initial stage (from green to two thirds of total moisture) of drying, the drying rates of presteamed specimens were faster than that of control specimens, and tended to increase as presteaming temperature increased (Fig. 3). First cause for the trend above is that the moisture diffusivity in the surface layers of presteamed specimens is increased due to the redistribution and partial removal of extractives from wood, some extractive being dissolved, and certain hydrolysable components being degraded during presteaming^10^. Increased moisture diffusivity favors increasing moisture evaporating. Second cause for the trend above is that faster drying results in larger tensile stresses in the surface layers of presteamed specimens compared to that in control specimens during the initial stage of drying. The larger tensile stresses and largely decreased MC (moisture content)s induce larger tensile creep deformation, which means that additional displacement and fixation occurs between the microfibrils inside the surface layers of presteamed specimens. This is the opposite direction from transverse shrinking. After the displacement, the distance between the microfibrils is increased because transverse shrinkage is decreased under larger tensile stresses. Therefore, the moisture transverse pathways in the surface layers of presteamed specimens become larger and more, which increases the drying rates of presteamed specimens during the initial stage of drying. The permeability of wood can be considered as an indicator of drying rate^11^. The increased permeability, which can be considered as a result of the increased distance between the microfibrils, inside the surfaces of presteamed specimens during initial drying stage (Fig. 1) and the faster drying rates of presteamed specimens during initial drying stage (Fig. 3) confirm the above explanation. As presteaming temperature increases, the tensile creep deformation becomes larger. Thus, the drying rates for presteamed specimens are the fastest for 100 °C the lowest for 60 °C and middle for 80 °C.

From the middle stage (from two thirds of total moisture to FSP) of drying, the drying rates of presteamed specimens became slower than that of control specimens, and tended to decrease as presteaming temperature increased (Fig. 3). After one third of total moisture is removed, the larger tensile stresses are reversed into larger compressive stresses in the surface layers of presteamed specimens, which is much earlier than control specimens^12^. The larger compressive stresses and decreased MCs induce larger compressive creep deformation, which means that additional displacement and fixation occur between the microfibrils in the direction of transverse shrinking. The distance between the microfibrils is reduced after the displacement. This is because transverse shrinkage is significantly increased under larger compression stresses. Therefore, the moisture transverse pathways in the surface layers become smaller and fewer. These pathways decrease the moving rates of the bound water within the cell walls, which moves by diffusion below the FSP, and the water vapor, which moves by diffusion in the lumens both above and below the FSP, from the inner part to the surface layers of presteamed specimens during middle drying stage. The decreased permeability, which can be considered as a result of the reduced distance between the microfibrils, inside the surfaces of presteamed specimens during middle drying stage (Fig. 1) and the slower drying rates of presteamed specimens during middle drying stage (Fig. 3) confirm the explanation above. As presteaming temperature increases, the compressive creep deformation becomes larger. Thus, the drying rates of presteamed specimens are the fastest for 60 °C the lowest for 100 °C and middle for 80 °C.

The crystallinity of the wood cellulose in the surface layers of presteamed samples increased during presteaming and continued increase during the initial and middle stage of drying (Fig. 2). This is because of the higher drying temperature^13,14^ and larger compressive stresses^13^. Increased crystallinity plays more obstructive role in moisture evaporating because water can not penetrate the crystalline cellulose^15^. The less amorphous region means the less moisture pathways in the surface layers. Thus, the lessened moisture pathways also decrease the drying rates of presteamed specimens during the middle stage of drying. The increased crystallinity in the surface layers of presteamed specimens (Fig. 2) supports the above explanation.

During the last drying stage (from FSP to final MC), the drying rates of presteamed specimens continued to decrease below that of control specimens (Fig. 3), and tended to decrease as presteaming temperature increased. As drying temperature rises, the surface layers are subjected to larger compressive stresses than that at the middle stage, as a result, the crystallinity is increased more. Consequently, the moisture transverse pathways in the surface layers of presteamed specimens become smaller and fewer than those at the middle drying stage, which further leads to decrease in the drying rates. The decreased permeability inside the surface layers during the last stage (Fig. 1) and the slower drying rates of presteamed specimens during last drying stage (Fig. 3) confirm the explanation above.

In summary, the larger and more moisture transverse pathways in the surface layers of presteamed specimens increase the drying rate during initial drying stage due to increased distance between the microfibrils. The smaller and fewer moisture transverse pathways in the surface layers decrease the moving rates of the bound water and water vapor from the inner part to the surface layers of presteamed specimens during the middle and last stage of drying because of reduced distance between the microfibrils and increased crystallinity. As a whole, average drying rate of presteamed specimens is slower than that of control specimens during whole drying period.

This finding is also significant for understanding the moisture moving mechanism of the other materials such as paper, paper-based packing materials, plant fiber materials, and wood reconstituted panels.

### Drying time

**Figure 4:**
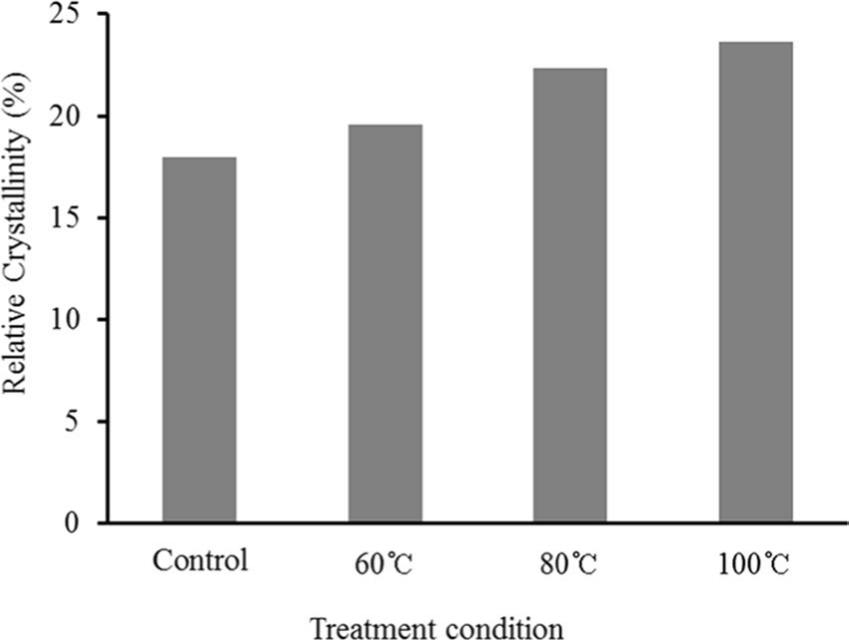
Drying curves of control and presteamed specimens.

The drying time of specimens from green to final MC was 17 days for control and at least 21 days for presteamed specimens (Fig. 4). This indicates that the drying time of presteamed oak lumber was delayed at least by 19 percent. This delay can be attributed to presteamed specimens’ slower drying rates from the middle to last stage of drying.

### Drying deformation

The F-test of drying deformation presented that there was significant difference in crooks (p < 0. 0402) and bow (p < 0.0115) among the specimens presteamed at 60 °C, the specimens presteamed at 80 °C, the specimens presteamed at 100 °C and control specimens, but there was no significant difference in cups (p < 0.1567) and twists (p < 0.1803)^16^.

Crook, which is a most serious defect because it wastes wood most of all deformations during further processing, and bow of presteamed specimen were significantly increased compared to that of control specimen (Table 1). This trend can be attributed to the larger compressive stresses after stresses reversed. This was agreement with the conclusion of Erickson^12^. Erickson found that the final compressive creep of the presteamed samples was 22 percent for flatsawn samples, and 34 percent for quatersawn samples, greater than that of the unsteamed samples^10^ when 1 inch red oak lumbers presteamed at 100 °C for 4 hours were dried. The larger compressive stresses at the last stage can easily induce the deformation of lumber. However, the difference in twist and cup between control and presteamed specimens was no significant (Table 1). This means that the presteaming did not significantly affect twist and cup. The reason for the phenomenon above is unknown. This is a subject that is necessary to be studied in the future.

**Table 1:** Deformation of oak lumbers after drying.

Treatment	Warp (mm)			
	Cup	Bow	Crook	Twist
Control		18 (1.00)	5.5 (0.00)	
60 °C	2.2 (0.21)	4.3 (0.50)	6.8 (1.01)	8.2 (0.00)
80 °C		6.0 (1.20)	7.3 (1.00)	15 (0.00)
100 °C	2.8 (0.00)	19.9 (3.50)	7.9 (1.20)	8.7 (2.20)

^*^The data inside bracket was STDEV.

## Methods

Flatsawn and quartersawn lumbers, 25.4 mm thick, 100 mm wide, and 1000 mm long, were sawn near to sapwood and at central part of green oak (*Quercus acutissima*.) logs with the diameter of 30 cm, respectively. The quartersawn lunbers were used to measure permeability and crystallinity while the flatsawn lumbers were used to measure the warp and checks of specimens. The number of specimens was 25 pieces for each treatment and 100 pieces for both quartersawn and flatsawn specimens, respectively. The green moisture content of specimens was 61.11 percent while the final MC of specimens was 11.36 percent.

The specimens were steamed at 60 °C 80 °C and 100 °C dry-bulb temperature and 0 °C wet-bulb depression for 4 hours in a forced-air drier (SKD-90HPT, Shinshiba, Asahikawa Japan) prior to kiln drying, respectively. Control specimens were stacked in the same drier and dried together with presteamed specimens using a drying schedule (Table 2), which was based on the schedules of most previous researchers in that presteaming was conducted at 100 °C dry-bulb temperature and 0 °C wet-bulb depression for 4 hours. The eight pieces of specimens, two specimens from each treatment, had been weighed at intervals of 24 hours to measure MCs during drying. At the initial, middle and last stage of drying, the quartersawn lumbers were pulled out of the drier. The thin lumbers with 100 mm long, 100 mm wide and 5 mm thick were sawn parallel to the wide surface layers of the quartersawn lunbers. From the thin lumbers, 120 samples with the diameter of 60 mm and thickness of 5 mm (10 samples from each treatment at each stage) were obtained in order to measure permeability and crystallinity. Once drying was complete, final MCs, the warp and checks of flatsawn lumbers were measured.

**Table 2:** Drying schedule.

Drying stage	Moisture content (%)	Dry-bulb temperature (°C)	Web-bulb depressions (°C)	Relative humidity (%)	Treatment time (h)
Presteaming		60.0, 80.0, 100.0	0.0	100	4
1	Above 40%	45.0	3.0	83	48
2	40–30	45.0	4.0	79	72
3	30–25	50.0	6.0	71	96
4			8.0	62	48
5	25–20	55.0	8.0	64	72
6	20–15	60.0	10.0	59	48
7	Below15%	60.0	14.0	46	72
8		65.0	14.0	48	48

The tangential permeability of samples was measured by a Capillary Flow Porometer (CFP-1200AEX, Porous Materials Inc., Ithaca U.S.A.) under the gaseous pressure of 1 bar after the lateral surfaces of samples were coated with quick adhesive. The crystallinity of the samples, oven-dried the samples used for measuring permeability in this study, was measured using a X-ray diffraction measuring device (XRO power, X’pert Pro Powder, PANaltical, Eindhoven Netherlands). At last, statistical analysis was conducted for the measured data using the SAS software.

## Supplementary information

Supplementary Information

## Data Availability

The data sets generated during and/or analysed during the current study are available from the corresponding author upon request.
