# Publisher Correction: Moisture transverse moving mechanism during presteamed oak lumber drying

**DOI:** 10.1038/s41598-020-74145-2

**Published:** 2020-10-07

**Authors:** Chengyuan Li, Chun-Won Kang, Xue-Feng Zhao

**Affiliations:** 1 Department of Wood Science and Engineering, Beihua University, Room 113, Yifu building, No. 3999, Binjiang East Road, Jilin City, Jilin Province 132013 P.R. China; 2 Department of Housing Environmental Design, and Research Institute of Human Ecology, College of Human Ecology, Chonbuk National University, Room 504, Building No. 7-1, 567 Baekje – daero, Deokjin-gu, Jeonju-si, Jeollabuk-do Republic of Korea; 3 Department of Wood Science and Engineering, Beihua University, Room 113, Yifu building, No. 3999, Binjiang East Road, Jilin City, Jilin Province 132013 P.R. China

Correction to: *Scientific Reports* https://doi.org/10.1038/s41598-019-54430-5, published online 03 December 2019

The original version of this Article contained an error in the order of the Figures, where Figures 1, 2, 3 and 4 were published as 3, 4, 2 and 1 respectively. The figure legends were listed in the correct order at time of publication.

This has now been corrected in the HTML and PDF versions of this Article.

