# Supplementary material for: Moisture transverse moving mechanism during presteamed oak lumber drying
Source: Sci Rep. 2019 Dec 3;9:18228. doi: 10.1038/s41598-019-54430-5 (PMC6890683; doi:10.1038/s41598-019-54430-5)
Supplement: Supplementary file 1 — Supplementary Information [file 41598_2019_54430_MOESM1_ESM.doc]

[**Supplementary Information**](https://static-content.springer.com/esm/art%3A10.1038%2Fs41598-019-46242-4/MediaObjects/41598_2019_46242_MOESM1_ESM.docx)

Moisture transverse moving mechanism during presteamed oak lumber drying

Author 1: Chengyuan Li

Author 2: Chun-Won Kang (corresponding author)

Author 3: Xue-Feng ZHAO

**Statistical test 1:**

**Name**: Permeability

**Result:**

There was significant difference in permeability among the specimens presteamed at 60℃, the specimens presteamed at 80℃, the specimens presteamed at 100℃ and control specimens at initial, middle and last drying stage (p<0.0001, ƞ^2^=0.98, ƞ_p_^2^=0.98 ).

**Statistical test 2:**

**Name**: Relative crystallinity

**Result:**

Result of one-way analysis of variance:

Difference in relative crystallinity among presteamed specimen at 60℃, presteamed specimen at 80℃, presteamed specimen at 100℃ and control specimen was significant (p<0.0001, ƞ^2^=0.95, ƞ_p_^2^=0.95)

Result of multiple comparison by LSD and Tukey method:

Comparison by LSD method indicated that difference in relative crystallinity between any two specimens among presteamed specimens at 60℃, presteamed specimens at 80℃, presteamed specimens at 100℃ and control specimens was significant. And the result of comparison by Tukey method was the same as that of LSD method.

**Statistical test 3:**

**Name**: Drying rate

**Result:**

There was significant difference in drying rates among the specimens presteamed at 100℃, the specimens presteamed at 80℃, and the specimens presteamed at 60℃ and control specimens during whole drying period (p<0.0003, ƞ^2^=0.98, ƞ_p_^2^=0.98) and initial (p<0.0001, ƞ^2^=0.99, ƞ_p_^2^=0.99 ), middle (p<0.0001, ƞ^2^=0.99, ƞ_p_^2^=0.99) and last drying stage (p<0.0001, ƞ^2^=0.99, ƞ_p_^2^=0.99).

**Statistical test 4:**

**Name**: Deformation (bow, cup, crook, twist)

**Result:**

There was significant difference in crooks (p<0.0402, ƞ^2^=0.08, ƞ_p_^2^=0.08) and bow (p<0.0115, ƞ^2^=0.10, ƞ_p_^2^=0.10) among the specimens presteamed at 60℃, the specimens presteamed at 80℃, the specimens presteamed at 100℃ and control specimens, but there was no significant difference in cups (p<0.1567, ƞ^2^=0.05, ƞ_p_^2^=0.05) and twists (p<0.1803, ƞ^2^=0.04, ƞ_p_^2^=0.04).

**Original data of statistical tests**

**Statistical test 1:** Permeability

data lcy2;

do temp=0 to 3;

do spm=1 to 10;

do stage=1 to 3;

input y@@;

output;

end;

end;

end;

cards;

35.59 35.05 27.78

35.09 34.02 26.95

35.28 34.01 27.89

34.49 34.03 28.01

35.37 34.01 27.42

34.98 34.03 27.49

34.69 32.98 27.29

34.80 34.09 26.93

34.97 33.89 25.95

35.92 34.19 26.98

35.80 28.42 25.13

36.06 32.22 25.55

35.52 30.55 26.09

36.04 30.24 29.10

36.20 31.01 26.87

35.78 29.79 27.35

36.02 31.01 25.98

37.01 30.07 26.67

36.08 31.05 26.58

36.01 30.53 27.04

36.69 26.48 23.10

38.76 27.09 23.01

38.09 26.14 22.89

37.98 26.06 25.02

38.02 25.98 23.01

37.99 26.77 21.87

38.58 25.39 23.02

36.98 26.57 22.92

38.98 27.01 23.33

37.97 28.01 22.90

44.22 25.31 22.74

42.87 26.22 23.03

43.90 25.98 21.95

44.82 25.97 23.09

45.31 24.98 22.98

44.96 25.08 22.88

43.90 23.94 25.09

44.32 25.89 24.44

42.98 23.98 22.80

44.89 25.76 21.99

;

Dependent Variable: y

Sum of

Source DF Squares Mean Square F Value Pr > F

Model 11 4851.512550 441.046595 744.72 <.0001

Error 108 63.960980 0.592231

Corrected Total 119 4915.473530

Source DF Type I SS Mean Square F Value Pr > F

temp 3 130.765957 43.588652 73.60 <.0001

stage 2 3727.710735 1863.855368 3147.17 <.0001

temp*stage 6 993.035858 165.505976 279.46 <.0001

Source DF Type III SS Mean Square F Value Pr > F

temp 3 130.765957 43.588652 73.60 <.0001

stage 2 3727.710735 1863.855367 3147.17 <.0001

temp*stage 6 993.035858 165.505976 279.46 <.0001

**Statistical test 2:** Relative crystallinity

data lcy1;

do rep=1 to 11;

do trt=0 to 3;

input y@@;

output;

end;

end;

cards;

17.62 19.56 22.86 22.66

17.73 18.87 21.18 23.99

18.48 20.33 23.01 24.16

17.92 19.97 22.10 23.90

18.00 20.13 22.20 24.12

17.44 18.98 21.98 23.96

17.92 19.90 23.10 23.68

17.99 18.98 21.98 23.10

18.44 19.89 22.29 22.98

17.92 19.00 23.01 23.81

17.95 19.56 22.37 23.64

;

proc glm data=lcy1;

class trt;

model y=trt;

means trt/lsd turkey;

run;


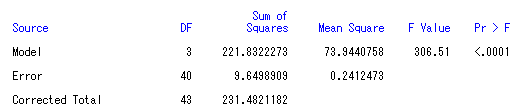


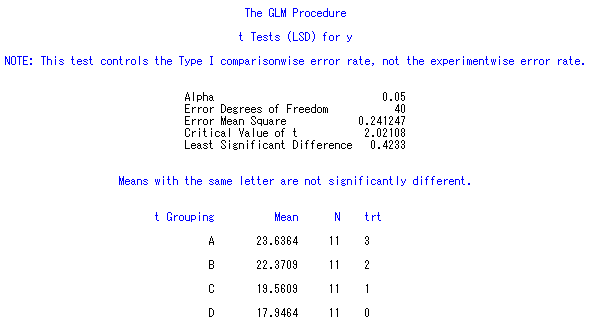


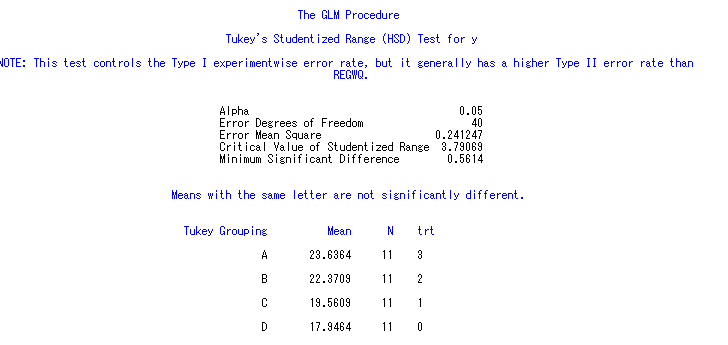


**Statistical test 3:** Drying rates

Average drying rate during whole drying period

| Control | 60℃ | 80℃ | 100℃ |
| --- | --- | --- | --- |
| 0.1070 | 0.0989 | 0.0925 | 0.0822 |
| 0.1090 | 0.0969 | 0.0905 | 0.0842 |

Sum of

Source DF Squares Mean Square F Value Pr > F

Model 3 0.00065762 0.00021921 109.60 0.0003

Error 4 0.00000800 0.00000200

Corrected Total 7 0.00066562

Drying rate during initial drying stage

| Control | 60℃ | 80℃ | 100℃ |
| --- | --- | --- | --- |
| 0.1312 | 0.1460 | 0.1604 | 0.1703 |
| 0.1292 | 0.1480 | 0.1584 | 0.1723 |

Sum of

| Source | DF | Squares | Mean Square | F Value | Pr > F |
| --- | --- | --- | --- | --- | --- |
| Model | 3 | 0.00185498 | 0.00061833 | 309.16 | <.0001 |
| Error | 4 | 0.00000800 | 0.00000200 |  |  |
| Corrected Total | 7 | 0.00186298 |  |  |  |

Drying rate during middle drying stage

| Control | 60℃ | 80℃ | 100℃ |
| --- | --- | --- | --- |
| 0.1237 | 0.1044 | 0.0853 | 0.0746 |
| 0.1217 | 0.1064 | 0.0833 | 0.0726 |

|  |  | Sum of |  |  |  |
| --- | --- | --- | --- | --- | --- |
| Source | DF | Squares | Mean Square | F Value | Pr > F |
| Model | 3 | 0.00287780 | 0.00095927 | 479.63 | <.0001 |
| Error | 4 | 0.00000800 | 0.00000200 |  |  |
| Corrected Total | 7 | 0.00288580 |  |  |  |

Drying rate during last drying stage

| Control | 60℃ | 80℃ | 100℃ |
| --- | --- | --- | --- |
| 0.0842 | 0.0731 | 0.0526 | 0.0469 |
| 0.0822 | 0.0751 | 0.0506 | 0.0449 |

|  |  | Sum of |  |  |  |
| --- | --- | --- | --- | --- | --- |
| Source | DF | Squares | Mean Square | F Value | Pr > F |
| Model | 3 | 0.00190332 | 0.00063444 | 317.22 | <.0001 |
| Error | 4 | 0.00000800 | 0.00000200 |  |  |
| Corrected Total | 7 | 0.00191132 |  |  |  |

**Statistical test 4:** Deformation (bow, cup, crook, twist)

**data** lcy;

do spm=**1** to **25**;

do temp=**0** to **3**;

input cup bow crook twist@@;

output;

end;

end;

cards;

0 0 0 0 0 0 5.2 0 0 0 6.3 0 0 25.2 0 0

0 0 0 0 0 0 0 0 0 0 0 0 0 0 9.9 0

0 0 0 0 0 0 0 0 0 0 0 0 0 23 0 0

0 0 0 0 0 0 0 0 0 0 0 15 0 18.2 0 0

0 0 0 0 2 0 0 0 0 0 0 0 0 0 0 8

0 0 0 0 2.4 0 0 0 0 7.6 0 0 0 0 6.9 0

0 0 0 0 0 0 0 0 0 0 0 0 0 0 7 0

0 0 0 0 0 0 0 0 0 0 0 0 0 14.1 0 0

0 0 0 0 0 4.9 0 0 0 0 0 0 0 0 6.7 0

0 19 0 0 0 0 0 0 0 5.3 0 0 2.8 0 0 0

0 0 0 0 0 0 0 0 0 0 0 0 0 0 7.6 0

0 0 0 0 0 4.4 0 0 0 0 6.1 0 0 20 0 0

0 0 0 0 0 0 0 0 0 0 7.8 0 0 0 7 0

0 17 0 0 0 0 0 0 0 0 0 0 0 0 9.5 0

0 0 0 0 0 0 0 0 0 0 0 0 0 20 0 0

0 0 0 0 2.3 0 0 0 0 0 8 0 0 19 0 0

0 0 0 0 0 4.2 0 0 0 0 8.2 0 0 0 0 9

0 0 0 0 0 0 8 0 0 4.8 0 0 0 0 8.2 0

0 0 5.5 0 0 0 7 0 0 0 8.3 0 0 0 9.9 0

0 0 0 0 0 0 7 0 0 6.4 0 0 0 0 0 5.1

0 0 0 0 0 0 7 0 0 0 0 0 0 0 0 3

0 0 0 0 0 3.7 0 0 0 0 0 15 0 0 0 5

0 0 0 0 0 0 0 8.2 0 0 0 0 0 0 0 5

0 18 0 0 0 0 0 0 0 0 6.5 0 2.8 0 0 0

0 0 5.5 0 0 0 0 0 0 0 0 0 0 0 0 0

;

**proc** **print**;

**run**;

**proc** **glm** data=lcy;

class temp;

model cup bow crook twist=temp;

lsmeans temp/pdiff e;

*estimate "control vs others" temp 3 -1 -1 -1/divisor=3 e;

**run**;

Result of F-test of cup;

|  |  | Sum of |  |  |  |
| --- | --- | --- | --- | --- | --- |
| Source | DF | Squares | Mean Square | F Value | Pr > F |
| Model | 3 | 1.53710000 | 0.51236667 | 1.78 | 0.1567 |
| Error | 96 | 27.68000000 | 0.28833333 |  |  |
| Corrected Total | 99 | 29.21710000 |  |  |  |

Result of F-test of bow;

|  |  | Sum of |  |  |  |
| --- | --- | --- | --- | --- | --- |
| Source | DF | Squares | Mean Square | F Value | Pr > F |
| Model | 3 | 378.805600 | 126.268533 | 3.88 | 0.0115 |
| Error | 96 | 3123.524000 | 32.536708 |  |  |
| Corrected Total | 99 | 3502.329600 |  |  |  |

Result of F-test of crook;

|  |  | Sum of |  |  |  |
| --- | --- | --- | --- | --- | --- |
| Source | DF | Squares | Mean Square | F Value | Pr > F |
| Model | 3 | 81.9467000 | 27.3155667 | 2.87 | 0.0402 |
| Error | 96 | 912.5352000 | 9.5055750 |  |  |
| Corrected Total | 99 | 994.4819000 |  |  |  |

Result of F-test of twist;

|  |  | Sum of |  |  |  |
| --- | --- | --- | --- | --- | --- |
| Source | DF | Squares | Mean Square | F Value | Pr > F |
| Model | 3 | 34.2411000 | 11.4137000 | 1.66 | 0.1803 |
| Error | 96 | 659.2800000 | 6.8675000 |  |  |
| Corrected Total | 99 | 693.5211000 |  |  |  |
